# IgG4-positive cell infiltration in various cardiovascular disorders - results from histopathological analysis of surgical samples

**DOI:** 10.1186/s12872-017-0488-3

**Published:** 2017-02-03

**Authors:** Ryoto Hourai, Satomi Kasashima, Koichi Sohmiya, Yohei Yamauchi, Hideki Ozawa, Yoshinobu Hirose, Yasuhiro Ogino, Takahiro Katsumata, Masahiro Daimon, Shu-ichi Fujita, Masaaki Hoshiga, Nobukazu Ishizaka

**Affiliations:** 1 Department of Cardiology, Osaka Medical College, Daigaku-machi 2-7, Takatsuki, Osaka 569-8686 Japan; 2 Department of Pathology, National Hospital Organization, Kanazawa Medical Center, Kanazawa, Japan; 3 Department of Thoracic and Cardiovascular Surgery, Osaka Medical College, Osaka, Japan; 4 Department of Pathology, Osaka Medical College, Osaka, Japan

**Keywords:** IgG4-related disease, Aortic valve, Aortic stenosis, Aortic wall, Aortic aneurysm

## Abstract

**Background:**

The diagnosis of Immunoglobulin G4 (IgG4)-related disease (IgG4-RD), in general, depends on serum IgG4 concentrations and histopathological findings; therefore, diagnosis of IgG4-RD in cardiovascular organs/tissues is often difficult owing to the risk of tissue sampling.

**Methods:**

Prevalence of IgG4-positive lymphoplasmacytic infiltration in 103 consecutive cardiovascular surgical samples from 98 patients with various cardiovascular diseases was analyzed immunohistochemically.

**Results:**

The diagnoses of the enrolled patients included aortic aneurysm (abdominal, *n* = 8; thoracic, *n* = 9); aortic dissection (*n* = 20); aortic stenosis (*n* = 24), aortic regurgitation (*n* = 10), and mitral stenosis/regurgitation (*n* = 17). In total, 10 (9.7%) of the 103 specimens showed IgG4-positive cell infiltration with various intensities; five of these were aortic valve specimens from aortic stenosis, and IgG4-positive cell infiltration was present at >10 /HPF in three of them. In one aortic wall sample from an abdominal aortic aneurysm, various histopathological features of IgG4-RD, such as IgG4-positive cell infiltration, obliterating phlebitis, and storiform fibrosis, were observed.

**Conclusions:**

IgG4-positive cell infiltration was observed in 9.7% of the surgical cardiovascular specimens, mainly in the aortic valve from aortic stenosis and in the aortic wall from aortic aneurysm. Whether IgG4-positive cell infiltration has pathophysiological importance in the development or progression of cardiovascular diseases should be investigated in future studies.

## Background

Immunoglobulin G4 (IgG4)-related disease (IgG4-RD), first documented by Hamano et al. in 2001 [1], is characterized by clinical (organ enlargement or nodular/hyperplastic lesions; serum IgG4 concentration ≥135 mg/dL) and histopathological (IgG4-positive cells infiltration, >10/HPF; IgG4 /IgG ratio, >40%) findings. IgG4-RD may affect a wide variety of organs concurrently or metachronously, [2, 3]. At present, IgG4-RD can be diagnosed either by comprehensive diagnostic criteria [4] or by organ specific diagnostic criteria [5–7], although there are no heart/artery-specific diagnostic criteria for IgG4-RD.

Several previous reports have demonstrated IgG4-RD in various-size arteries and pericardium [8–13]. Most, but not all, cases of IgG4-RD in cardiovascular organs have diagnosed on the basis of histopathological findings in samples obtained either at the time of surgery or by autopsy. Biopsy of these vital organs in other situations is generally associated with considerable risk; therefore, IgG4-RD in cardiovascular organs may be underdiagnosed. In the current study, we have investigated the prevalence and the extent of IgG4-positive lymphoplasmacytic infiltration observed in tissue samples of various cardiovascular diseases obtained at the time of surgery.

## Methods

### Histology specimens and patients

In this study, histological analysis was carried out on 103 consecutive histologic specimens obtained from 98 patients who underwent cardiovascular surgery at Department of Thoracic and Cardiovascular Surgery, Osaka Medical College between January 2014 and December 2014. None of the enrolled patients had been diagnosed with or was suspected to have IgG4-RD at the time of surgery. Due to the retrospective design of the current study, serum IgG4 concentrations of the enrolled patients at the time of surgery were not available. Histological evaluation was carried out on formalin-fixed and paraffin embedded specimens. Immunostaining was performed with mouse monoclonal antibody against IgG4 (MC011, Biding Site, Birmingham, UK). IgG staining was performed with anti-IgG antibody (DAKO, Glostrup, Denmark). For some specimens, owing to the high background of IgG staining, anti-human CD138 antibody (AbD, Serotec, Oxford, UK) was used to stain plasma cells. In specimens where IgG4-positive cell infiltration was observed, we counted three ×40 fields with the highest number of IgG4-positive plasma cells, and then calculated the average number of IgG4-positive plasma cells within these fields. For the purpose of calculating the IgG4-to-IgG, or IgG4-CD138, ratio, the same three fields were counted [14].

## Results

### Patients and histology samples

In total, 103 histological samples from 98 patients were analyzed. About one fifth of the patients had a history of malignancy, and nine patients were undergoing chronic hemodialysis (Table 1). The most common diagnosis among the cardiovascular conditions was aortic stenosis (24 patients), followed by aortic dissection (20 patients) (Table 2). Aortic aneurysm, either abdominal (*n* = 8) or thoracic (*n* = 9), was diagnosed for 17 patients. There were no patients who were diagnosed with inflammatory aortic aneurysm. From patients with either mitral stenosis (*n* = 4) or mitral regurgitation (*n* = 7), 11 mitral valve samples and 6 myocyte samples were obtained. Five tumors were analyzed, comprising atrial myxoma (*n* = 3), thymoma (*n* = 1), and pulmonary artery sarcoma (*n* = 1).

**Table 1:** Baseline characteristics of the study patients

No. of subjects	98
Male gender, n (%)	58 (59.2)
Age, years	68.9 ± 11.7
BMI, kg/m^2^	22.4 ± 3.6
Chronic hemodialysis, n (%)	9 (9.2)
Co-morbidities	
Hypertension, n (%)	62 (63.3)
Diabetes, n (%)	25 (25.5)
Collagen vascular diseases, n (%)	5 (5.1)
Malignant disorders, n (%)	20 (20.4)
Smoking status	
Never (%)	48 (49.0)
Former (%)	41 (41.8)
Current (%)	9 (9.2)
Laboratory data	
White blood cell count, ×10^3^/μL	6.5 ± 3.4
Eosinophil count, /μL	188 ± 154
Hemoglobin, g/dL	12.6 ± 2.0
Platelet count, ×10^4^/μL	19.8 ± 5.9
Total protein, g/dL	6.8 ± 0.6
Albumin, g/dL	3.8 ± 0.5
Creatinine^a^, mg/dL	1.0 ± 0.4
eGFR^a^, mL/min/1.73 m^2^	59.2 ± 20.0
C-reactive protein, median (IQR) mg/dL	0.14 (0.04–1.65)
Amylase, U/L	90.9 ± 42.7

^a^Excluding those undergoing chronic hemodialysis. IQR indicates interquartile range. Data indicate mean ± standard deviation unless otherwise described

**Table 2:** Number and type of sampled cardiovascular tissues by clinical diagnosis

		Sampled tissues							
Clinical diagnosis	Pt. no.	Aortic wall	Arterial wall	Aortic valve	Mitral valve	Myocardium	Pericardium	Tumor	Thrombus
Abdominal aortic aneurysm	8	8							
Thoracic aortic aneurysm	9	9							
Aortic dissection (type A)	15	15							
Aortic dissection (type B)	5	5							
Annuloaortic ectasia	1	1							
Annuloaortic ectasia + aortic regurgitation	3	3		3					
Iliac arterial aneurysm	1		1						
Aortic stenosis	22			22					
Aortic regurgitation	4			4					
Aortic stenosis + regurgitation	2			2					
Aortic regurgitation + mitral stenosis	1			1	1				
Mitral stenosis	2				2				
Mitral regurgitation	11				5	6			
Mitral stenosis + regurgitation	2				2				
Mitral stenosis + mediastinal tumor	1				1			1	
Pericarditis	3						3		
Atrial myxoma	3							3	
Intra-pulmonary arterial tumor	1							1	
Pulmonary thrombosis	1								1
Arteriosclerosis obliterans	2	2							
Right aortic arch	1	1							
	Total	Total of tissues sampled (103 sampled regions)							
	98 pts.	44	1	32	11	6	3	5	1

### Prevalence of IgG4-positive cell infiltration in cardiovascular samples

Of the 103 histological samples, IgG4-positive cell infiltration was demonstrated immunohistochemically in 10 samples (10 patients): five in aortic valve samples, and five in aortic wall specimens (Table 3). Of note, all five aortic valve specimens that showed positive IgG4-cell infiltration were from patients with aortic stenosis; therefore the prevalence of of IgG4-positive cell infiltration positivity in aortic valve samples from aortic stenosis was as calculated to be 21% (5/24). The prevalence of IgG4-positivity in the cardiovascular specimen tended to be higher among patients with aortic stenosis (21%) than among patients without aortic stenosis (5/74, 7%, *P* = 0.062 by Fisher’s exact test).

**Table 3:** Clinical and histopathological findings of the 10 cases with IgG4-positive cell infiltration

Case no.	Case 1	case 2	Case 3	case 4	Case 5	Case 6	Case 7	Case 8	Case 9	Case 10
Sex	male	female	female	male	female	female	female	male	female	male
Age	67	93	72	66	56	84	74	67	64	63
Cardiovascular disorder	Abdominal aortic aneurysm	Aortic dissection (type A)	Aortic dissection (type A)	Thoracic aortic aneurysm	Abdominal aortic aneurysm	Aortic stenosis	Aortic stenosis + regurgitation	Aortic stenosis + regurgitation	Aortic stenosis	Aortic stenosis
Tissue sampled	aortic wall	aortic wall	aortic wall	aortic wall	aortic wall	aortic valve	aortic valve	aortic valve	aortic valve	aortic valve
IgG4 (/HPF)	101	18.7	35	37.5	5.2	73	3	20	3	23.5
IgG4/IgG (CD138) ratio (%)	75	41.3	46.7	51.3	12.4	44.8^a^	8^a^	17.4^a^	30^a^	31.5^a^
Obliterating phlebitis	+	-	-	-	-	-	-	-	-	-
Storiform fibrosis	+	-	-	+	-	-	-	-	-	-
Lymphoid follicle	+	-	-	+	+	-	-	-	-	-
Eosinophil infiltration	+	-	-	-	-	-	-	-	-	-
Neutrophil infiltration	-	+	-	-	-	-	-	-	-	-
Perineural inflammation	+	-	-	-	-	-	-	-	-	-
Granuloma	-	-	-	-	-	-	-	-	-	-
Thickness of adventitial fibrosis, mm	2.4	1	2	1.2	0.1	N/A	N/A	N/A	N/A	N/A
Hemosiderin deposition	-	+	+	-	+	+	+	-	-	+

*N/A* Not assessable

^a^CD138 staining was performed in the place of IgG staining

Three of the 10 patients with a histological sample showing IgG4-positive cell infiltration were undergoing chronic hemodialysis; this prevalence of hemodialysis (30%) was significantly higher than that among patients without IgG4-positive cell infiltration (6/88, 7%; *P* = 0.047 by Fisher’s exact test). IgG4-positive cell infiltration was not observed in any of the mitral valve samples (*n* = 11), myocardium samples (*n* = 6), cardiac tumors (*n* = 3), pericardium samples (*n* = 3), mediastinal thymoma (*n* = 1), or pulmonary artery sarcoma (*n* = 1).

### IgG4-positive cell infiltration in aortic valve specimens

Five of 24 aortic valve specimens showed IgG4-positive cell infiltration; therefore, the ratio of IgG4-positive infiltration among patients with aortic stenosis was calculated to be 21% (Table 3). The stenosed aortic valves associated with IgG4-positive cell infiltration were tricuspid in four patients and bicuspid in one patient (case 10), and all these stenosed aortic valves were atherosclerotic (i.e., non-rheumatic) in nature.

**Fig. 1:**
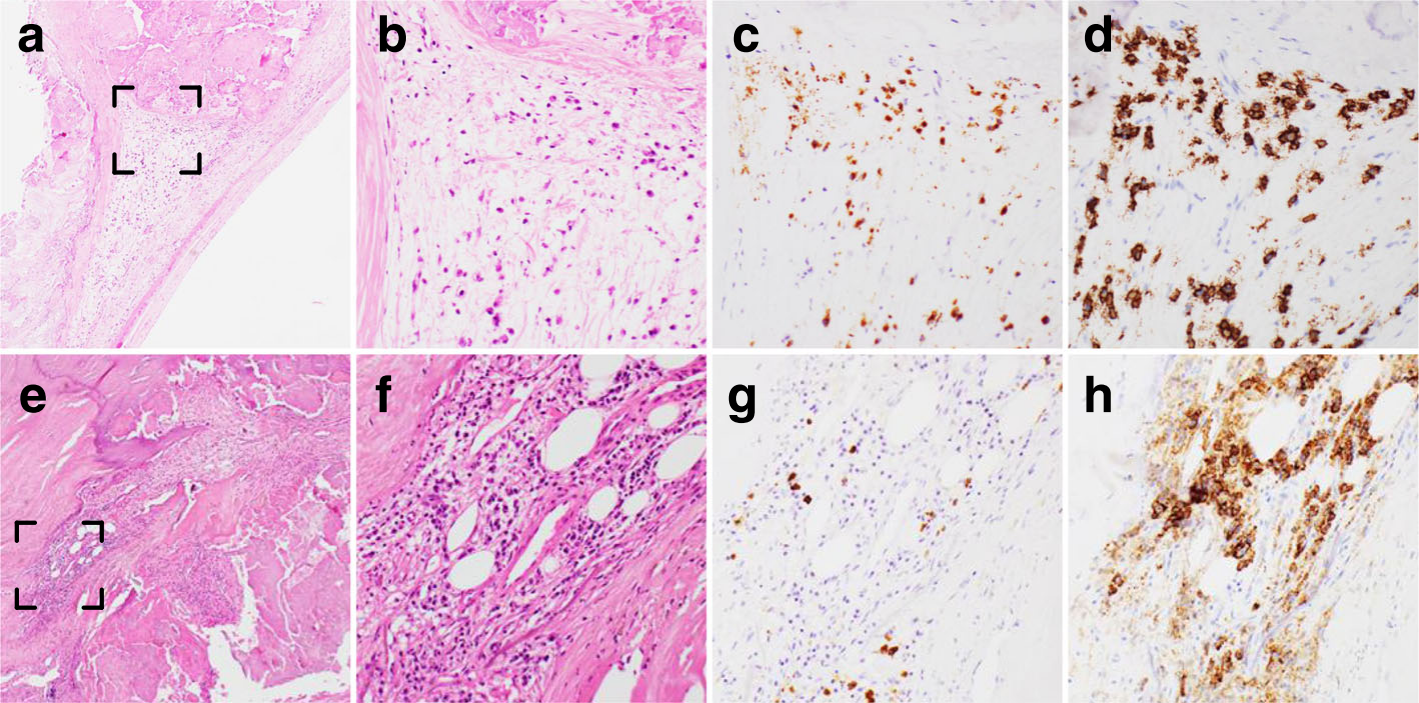
Histopathological findings of tissue samples obtained from two patients with aortic stenosis (case 6 and case 8). **a**-**d** Case 6. **e**-**h** Case 8. **a**, **b**, **e**, **f** Hematoxylin and eosin (HE) staining. **c**, **g** IgG4 staining. **d**, **h** CD138 staining. **b** Higher magnification image of the bracketed area in **a**. The number of IgG4-positive cells was 73/HPF and the IgG4/CD138 ratio was 45%. **f** Higher magnification image of the bracketed area in **e**. Original magnification, ×40 (**a**, **e**), and × 200 (**b**-**d**, **f**-**h**). The number of IgG4-positive cells was 20/HPF and IgG4/CD138 ratio was 17%

The histopathological findings in two cases (case 6 and case 8) are demonstrated in Fig. 1. In case 6, an 84-year female patient, the IgG4/CD138 ratio was greater than 40% (Figs. 1a-d). In case 8, a 67-year male patient, the sample showed abundant infiltration of CD138-positive (CD138+) plasma cells; however, only a minor proportion of them were IgG4-positive (IgG4-positive/CD138+, <20%) (Figs. 1e-h). Based on the low IgG4-positive/CD138+ ratio and absence of characteristic histologic features, such as obliterating phlebitis and storiform fibrosis, none of the five aortic valve specimens with IgG4-positive cell infiltration (cases 6–10) was considered to indicate IgG4-RD.

According to clinical records, during the median follow-up period of 464 days (range, 316–636 days), neither occurrence of IgG4-RD nor dysfunction of the prosthetic valve (one bioprosthetic and four mechanical) was noted in any of the five patients with positive IgG4-positive cell infiltration in their aortic valve sample.

### IgG4-positive cell infiltration in aortic wall specimens

**Fig. 2:**
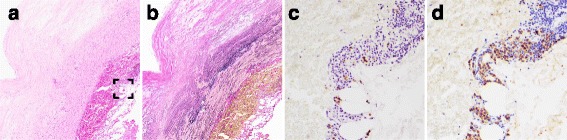
Histopathological findings of tissue samples obtained from patients with thoracic aortic aneurysm and suspected reactive inflammatory cell infiltration (case 4). **a** HE staining. **b** Elastica van Gieson staining. **c** IgG4 staining. **d** IgG staining. **c** and **d** show higher magnification images of the bracketed area in A. Original magnification, ×40 (**a**, **b**), and × 200 (**c**, **d**)

The remaining five samples with IgG4-positive cell infiltration were from patients with abdominal aortic aneurysm (AAA, *n* = 2), aortic dissection (*n* = 2), and thoracic aortic aneurysm (TAA, *n* = 1) (Table 3). The prevalence of IgG4-positive cell infiltration among patients with aortic aneurysm and aortic dissection was thus calculated to be 20% (3/15) and 10% (2/20), respectively. In case 4, the specimen showed IgG4-positive plasmacytic infiltration with an IgG4-positive/IgG-positive ratio above 40% but obliterating phlebitis was not observed; therefore, the suspected diagnosis was reactive infiltration of lymphoid cells with IgG4-positive cell infiltration (Fig. 2).

**Fig. 3:**
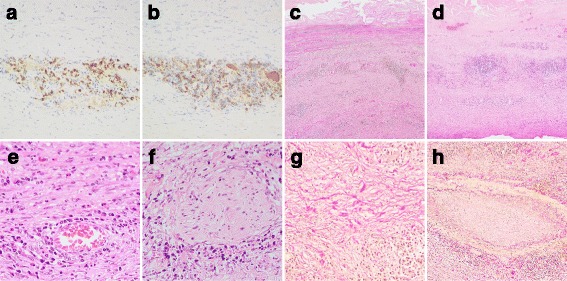
Histopathological findings of tissue samples from case 1. **a** IgG4 staining. **b** IgG staining. **c**, **g**, **h** Elastica van Gieson staining. **d**-**f**, HE staining. **a**, **b** The IgG4-positive/IgG-positive ratio was >70%. **c**, **d** Prominent adventitial thickening (**c**) with formation of lymphoid follicles (**d**) were observed. **e** Infiltration of eosinophils. **f** Perineural inflammatory cell infiltration. **g** Storiform fibrosis. **h** Obliterative phlebitis. Original magnification, ×200 (**a**, **b**, **g**), ×40 (**c**, **d**), ×400 (**e**, **f**), and × 100 (**h**)

**Fig. 4:**
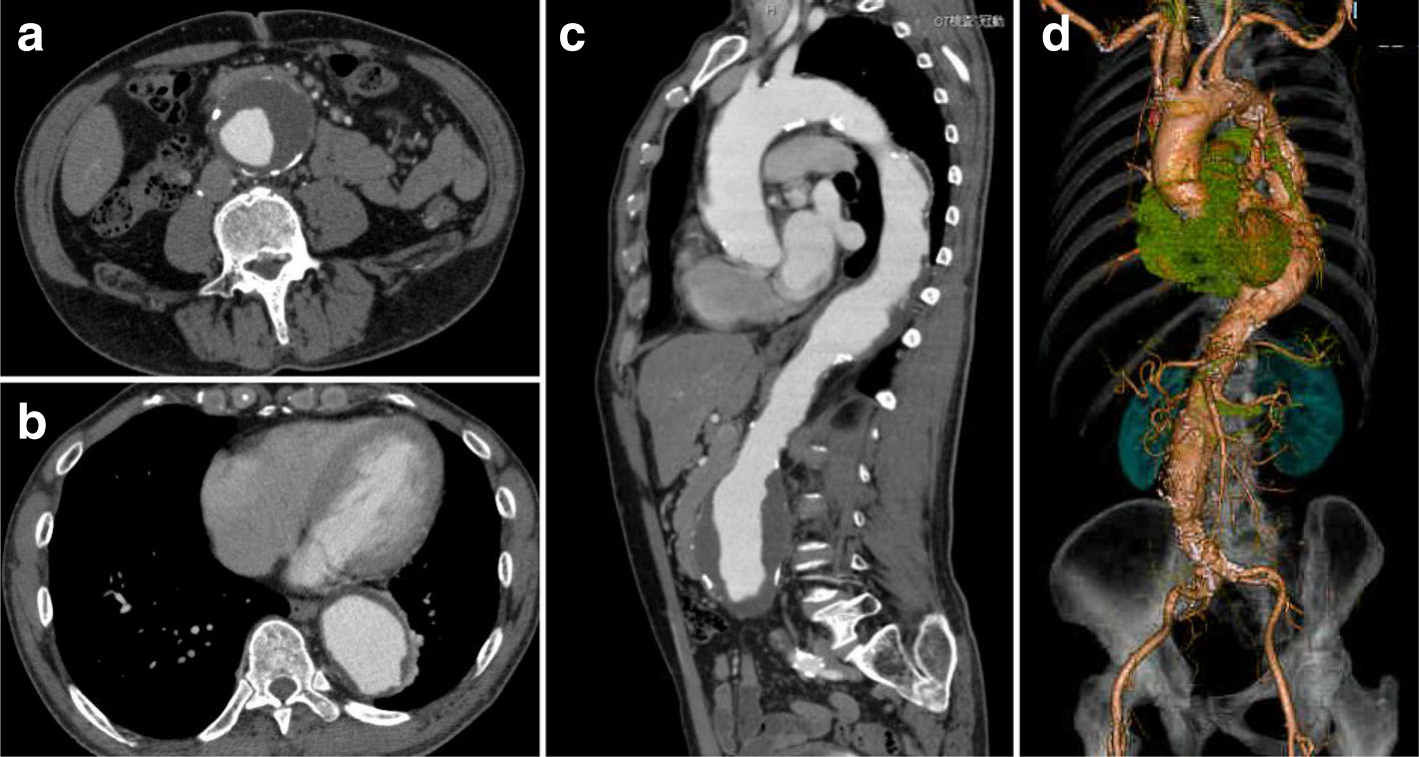
Contrast enhanced computed tomography of case 1 before the operation for abdominal and thoracic aortic aneurysm. **a**, **b** Axial view. **a** Aneurysmal dilatation with intraluminal thrombus was observed in the abdominal aorta. **b** The thoracic aorta was also enlarged. **c**, **d** Sagittal reconstruction (**c**) and 3-dimensional reconstruction (**d**), demonstrating the morphology of the thoracic and abdominal aorta and branches

The sample of case 1, who was diagnosed with AAA, showed not only IgG4-positive cell infiltration, but also other pathological features that are characteristic of IgG4-RD, such as adventitial thickening, lymphoid follicle formation, eosinophil infiltration, perineural infiltration, storiform fibrosis, and obliterating phlebitis (Fig. 3). Based on these observations, IgG4-RD was strongly suggested histopathologically in this patient. The patient also had TAA (Fig. 4) and underwent thoracic endovascular aortic repair 8 months after surgery for the AAA.

According to clinical records, during the median follow-up of 367 days (range, 50–795 days), occurrence of IgG4-RD in tissues other than cardiovascular tissues was not noted in any of the five patients with positive IgG4-positive cell infiltration in the aortic wall samples. In addition, in case 1, neither clinically significant systemic inflammation nor graft problems occurred during the follow up period.

## Discussion

In the current study, whether, and if so to what extent, IgG4-positive cell infiltration was present in 103 consecutive surgical samples from 98 patients undergoing cardiovascular surgery. In total, IgG4-positive cell infiltration was observed in 10 histological samples. IgG4-positive cell infiltration was most frequently observed in aortic wall of the AAA (3/8, 38%) followed by the aortic valve of aortic stenosis (5/24, 21%), and the aortic wall of dissecting aneurysm (2/20, 10%). On the other hand, IgG4-positive cell infiltration was not observed among the remaining 93 histological specimens. These findings indicate that IgG4-positive cell infiltration may occur in certain limited cardiovascular disease. In one patient with AAA (case 1), in addition to IgG4-positive cell infiltration, other histopathologic features characteristic of IgG4-RD listed in the International Consensus Diagnostic Criteria (ICDC) [15] were observed, such as storiform fibrosis and obliterating phlebitis (Fig. 3), leading to the increased possibility of an IgG4-related aortic lesion.

There are several previous studies examining the prevalence of IgG4-RD or IgG4-positive lymphoplasmacytic infiltration in cardiovascular histological specimens. Kasashima et al. reported that 13 (5.2%) of 252 surgically-treated AAA cases [8] and 5 (7.0%) of 71 surgically-treated TAA cases [16] may have belonged to IgG4-RD. In the current study, if case 1 were diagnosed with IgG4-RD, the prevalence of undiagnosed IgG4-RD among AAA might be calculated as 1/8 (13%). It should be noted, however, that elevation of serum IgG4 levels-information that was not available for this patient-is indispensable for the definitive diagnosis of IgG4-RD [4, 8, 17]. Regarding other organ-specific diagnostic criteria, such as those targeting IgG4-related sialodacryoadenitis and autoimmune pancreatitis, it may be possible to diagnose IgG4-RD without serum IgG4 levels; however, there are currently no organ-specific diagnostic criteria for IgG4-related cardiovascular lesions in Japan, Thus, what we showed in the current study was not the prevalence of IgG4-related disease, but the prevalence of IgG-positive cell infiltration in cardiovascular surgical samples.

Lymphocytic infiltration in calcified aortic stenosis has been reported in several previous studies. Wallby et al. showed that infiltration of T lymphocytes and plasma cells has been frequently observed in non-rheumatic stenosed tricuspid or bicuspid aortic valves [18]. In addition, Wu et al. proposed that lymphocytic infiltration may not represent a secondary response to inflammation, but may provide components of the valvular injury responsible for aortic stenosis [19]. There are also a few reports regarding IgG4-positive cell infiltration in aortic stenosis. Steiner et al. reported that 13 (87%) of 15 stenosed aortic valve samples showed IgG4-positive cell infiltration [20]. In their study, 15 stenosed aortic valve samples were selected from a total of 178 aortic valve samples by the presence of intense cellular infiltration (≥100 cells/HPF); therefore, the true percentage of samples with IgG4-positive cell infiltration among the overall (i.e., 178) cases of aortic stenosis could not be determined. It should be noted that in the current study, we did not select aortic specimens with intense cellular infiltration (≥100 cells/HPF) before IgG4-staining; therefore, we cannot simply compare the prevalence of IgG4-cell infiltration in stenosed aortic valve samples between Steiner et al.’s study and ours.

Chronic hemodialysis was more prevalent among patients with histological samples showing IgG4-positive cell infiltration. Krediet et al. reported that serum IgG4 subclass was decreased among those undergoing ambulatory peritoneal dialysis and was not significantly different among those undergoing hemodialysis as compared with healthy volunteers [21]. Although the possibility exists that IgG4-related kidney disease might have preceded the introduction of hemodialysis [22] for some patients in whom IgG4-positive cell infiltration was shown, whether there is a causal or resultant relationship between these conditions awaits further large-scale studies.

There are some limitations in the current study. First, due to the study design, data on serum IgG4 concentrations at the time of surgery were not available. Second, owing to the cross-sectional nature of the study, the pathophysiological importance of IgG4-positive cell infiltration in cardiovascular tissues cannot be addressed. Whether cardiovascular patients with IgG4-positive cell infiltration have a different clinical course [23] and an altered responsiveness to drug therapy as compared with those without IgG4-positive cell infiltration should be analyzed in future longitudinal studies. Third, IgG4-positive lymphoplasmacytic infiltration is not a feature exclusive to IgG4-RD; it may be observed in several immune and/or inflammatory disorders such as Castleman disease [24], Rosai-Dorfman disease [25], Wegener granulomatosis [26], and sialadenitis caused by sialolithiasis [27]. Lastly, we examined the presence or absence of IgG4-positive cell infiltration in one slide for each of the 103 samples; therefore, the true prevalence of IgG4-positive cell infiltration among these surgical samples might have been greater than what we have observed. The strength of the current study, on the other hand, was that by means of a comprehensive histopathological analysis, we have been able to estimate the prevalence and extent of IgG4-positive cell infiltration in various cardiovascular diseases.

## Conclusion

In conclusion, by analyzing 103 consecutive surgical samples obtained from 98 patients undergoing cardiovascular surgery, IgG4-positive cell infiltration was noted in 5 (21%) cases of 24 aortic stenosis and 5 cases of aortic aneurysm or aortic dissection. These findings collectively indicate that IgG4-positive cell infiltration is not a rare finding in cardiovascular diseases, especially in aortic stenosis, aortic aneurysm, and aortic dissection. The pathophysiological importance of IgG4-positive infiltration in these disorders should be investigated in further studies.

## Abbreviations

AAA: Abdominal aortic aneurysm
IgG4: Immunoglobulin G4
IgG4-RD: IgG4-related disease
TAA: Thoracic aortic aneurysm
